# A Mini Review on Nanocarbon-Based 1D Macroscopic Fibers: Assembly Strategies and Mechanical Properties

**DOI:** 10.1007/s40820-017-0151-7

**Published:** 2017-08-16

**Authors:** Liang Kou, Yingjun Liu, Cheng Zhang, Le Shao, Zhanyuan Tian, Zengshe Deng, Chao Gao

**Affiliations:** 1 Shaanxi Coal and Chemical Technology Institute Co., Ltd, 2 Jinye Road 1, Xi’an, 710070 People’s Republic of China; 2 MOE Key Laboratory of Macromolecular Synthesis and Functionalization, Department of Polymer Science and Engineering, Zhejiang University, Hangzhou, 310027 People’s Republic of China; 3 State Key Laboratory for Modification of Chemical Fibers and Polymer Materials, Donghua University, Shanghai, 201620 People’s Republic of China

**Keywords:** One dimensional, Macroscopic architectures, Carbon nanotubes, Graphene fibers, Assembly strategies, Mechanical performance

## Abstract

Nanocarbon-based materials, such as carbon nanotubes (CNTs) and graphene have been attached much attention by scientific and industrial community. As two representative nanocarbon materials, one-dimensional CNTs and two-dimensional graphene both possess remarkable mechanical properties. In the past years, a large amount of work have been done by using CNTs or graphene as building blocks for constructing novel, macroscopic, mechanically strong fibrous materials. In this review, we summarize the assembly approaches of CNT-based fibers and graphene-based fibers in chronological order, respectively. The mechanical performances of these fibrous materials are compared, and the critical influences on the mechanical properties are discussed. Personal perspectives on the fabrication methods of CNT- and graphene-based fibers are further presented.

## Introduction

Nanocarbon-based materials, such as carbon nanotubes (CNTs) and graphene, are extensively investigated due to their outstanding properties such as large theoretical specific area, high Young’s modulus (*E*), unparalleled thermal and electricity conductivity, and ultrahigh electron mobilities [1–4]. It is of practical significance to integrate the superior physical and chemical properties of individual CNTs or graphene to the macroscopic level by assembling them into macroscopic architectures [4]. As a representative of high-performance fibers, carbon fibers featured by their high tensile strength (*σ*) and *E* are widely used in aerospace, aviation, automobile, electronic, mechanical, chemical, textile, and other civilian industry. However, the electrical and thermal conductivities of carbon fibers are not satisfied. One-dimensional (1D) macroscopic architecture of CNTs and graphene, namely CNTs fibers (CFs) and graphene fibers (GFs), are analogous to carbon fibers in constitution and structure. Moreover, the combined performances including good flexibility and excellent electrical and thermal conductivities make them potential as structural–functional integrated materials. As more attentions have been attracted to the macroscopic fibers of 1D CNTs and 2D (two-dimensional) graphene, it is the time to give a correlative review about CFs and GFs together. We think that this insightful review can help researchers to have a better systematic understanding of the whole developing journey of macroscopic assembled fibers. In this mini review, we firstly introduce the assembly strategies of CFs and GFs and then compare the mechanical performance of fibers made from different approaches. Finally, we present our perspectives on the future of CFs and GFs.

## CNTs Fibers: 1D CNTs Macroscopic Architectures

Macroscopic fibers containing only CNTs will yield great advances in high-tech applications (e.g., aerocraft, lightweight cables) if they can attain the extraordinary mechanical and electrical properties of individual CNTs. This requires that the CNTs in the fibers are sufficiently long, highly aligned, and packed in an arrangement, and is nearly free of defects. Until now, the strategies for assembling CNTs to CFs can be classified into liquid- or solid-state spinning approaches. Liquid-state spinning approaches involve the dispersing of CNTs in a suitable solvent and shaping in coagulation bath. Solid-state spinning approach circumvents the dissolution problem either by drawing a fiber from a vertically grown CNT array or by drawing directly from an aerogel in the furnace [5].

### Liquid-State Spinning Approaches

Liquid-state spinning approaches generally include polymer-based coagulation spinning method, liquid crystalline (LC) spinning method, polymer-free spinning method, and dip-coating method. Among them, polymer-based coagulation spinning method is first introduced to achieve CFs.

#### Polymer-Based Coagulation Spinning Method

**Fig. 1:**
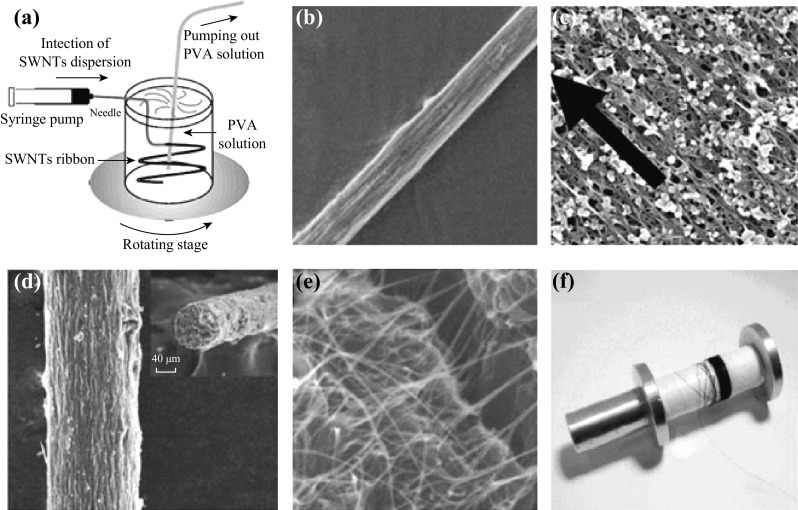
**a** Schematic of the experimental setup for spinning CFs using polymer-based coagulation spinning method. SEM images of **b** a CF and **c** a ribbon (*black arrow* indicates the main axis) deposited on a substrate. SEM images of DNA-SWNT fibers **d** (The insert image show the round cross section) and **e** a magnified image of the cross section of an SWNT bundle coated with DNA. **f** A 10-m-long MWNT fiber collected on a small winder. **a**–**c** Reproduced with permission from Ref. [6]. Copyright 2015, American Association for the Advancement of Science. **d**, **e** Reproduced with permission from Ref. [8]. Copyright 2008, Wiley–VCH. **f** Copyright Reproduced with permission from Ref. [11]. Copyright 2005, American Chemical Society

The history of CFs fabrication dates back as far as 17 years ago. In 2000, Poulin’s group dispersed single-wall carbon nanotubes (SWNTs) in sodium dodecyl sulfonate (SDS) solutions, recondensed the nanotubes in the flow of poly(vinyl alcohol) (PVA) solution to form a nanotube mesh, and then collated this mesh to a nanotube fiber (Fig. 1a–c). When SWNTs dispersion was slowly injected through a syringe needle or a thin glass capillary, flow-induced alignment of the nanotubes occurred in the direction of the fluid velocity. The flow-induced alignment was maintained by the PVA solution since it allows the SWNTs to be rapidly stuck together. The as-made CNT/PVA fibers possess good mechanical performance with 150 MPa for *σ* and 15 GPa for *E* [6]. Replaced SDS surfactant with DNA, the CFs were also mechanically strong and conductive [7]. Scanning electron microscopy (SEM) images reveal that the fiber has a circular cross section with 80 μm in diameter (Fig. 1d) and well-resolved network of DNA-SWNT hybrid (Fig. 1e) [8].

By utilizing SWNTs synthesized from carbon monoxide and lithium dodecyl sulfate as a surfactant, Baughman’s groups first produced mechanically strong gel fibers. After post-treatment with acetone-washing bath, continuous, multifunctional dry CFs were achieved. The resultant CFs showed a dramatically increased *σ* and toughness (570 J g^−1^) [9, 10] Besides, hot-drawing technique was utilized to improve the properties of CFs [11]. The resulting 10-m-long fiber collected onto a small winder is shown in Fig. 1f. This treatment induced a crystallinity increase in the PVA and an unprecedented degree of alignment of the CNTs, leading to a markedly improved energy absorption at low strain [11].

#### LC Spinning Method

**Fig. 2:**
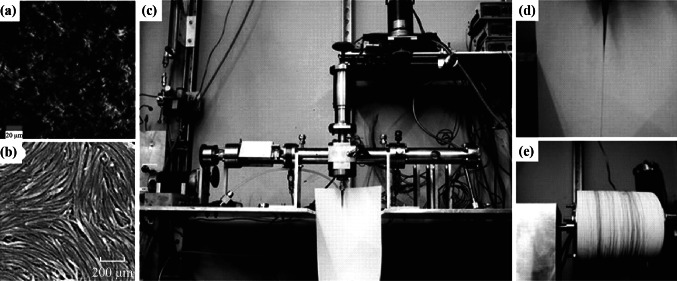
**a** Polarizing optical microscopy (POM) observation of the SWNTs dispersed in 123% sulfuric acid. **b** Field emission gun scanning electron microscope (FEGSEM) image of **a** −1/2 disclination in the nematic phase for CNTs. **c** The apparatus used for mixing and extruding neat SWNT fibers. **d** A jet of SWNT dispersion being extruded from a capillary tube. **e** A 30-m-long spool of water-coagulated SWNT fiber. **a**, **b** Reproduced with permission from Ref. [13, 14]. Copyright 2006, American Chemical Society. **c**–**e** Reproduced with permission from Ref. [18]. Copyright 2004, American Association for the Advancement of Science

The main challenge of producing CFs is to disperse the CNTs at high concentration for efficient alignment. Using surfactant is an effective approach to disperse CNTs into solutions, yet there is a loss in the properties of CFs due to the decreased van der Waals interaction between the neighboring CNTs. So super-acid (100% sulfuric acid) was introduced to increase the dispersity of CNTs. The protonated SWNTs in the super-acid behave as rigid-rod-like liquid crystals (LC) and exhibit an isotropic-to-nematic phase transition with an increased concentration (Fig. 2a, b) [12–14]. Based on this behavior, LC spinning technique was proposed to produce highly orientated CF [15, 16]. Windle’s group spun fibers consisting of multi-wall carbon nanotubes (MWNTs) directly from the lyotropic LC phase. Ethylene glycol was used as the lyotropic solvent, enabling a wider range of CNTs types (SWNTs, DWNTs, MWNTs) to be spun than previously. Fibers spun with CNTs and nitrogen-doped CNTs were also compared [16]. The spun fibers were flexible and can be easily twisted and knotted. The orientation increased with decreasing the diameter of fibers, leading to better thermal and electrical conductivity [17].

Ericson et al. [18] improved this method by dispersing SWNTs in fuming sulfuric acid (102% sulfuric acid), which charges SWNTs by acid anions and orders them into an aligned phase. When the viscosity reached a steady state, the SWNTs dispersion was extruded through a small capillary tube into a coagulation bath and collected onto a Teflon drum after washing (Fig. 2c–e). The resulting CFs possess good mechanical (120 GPa for *E* and 116 MPa for *σ*) and electrical (5000 S cm^−1^) properties.

#### Polymer-Free Spinning Method

**Fig. 3:**
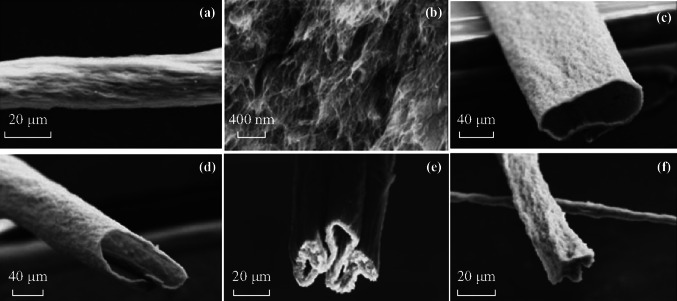
SEM images of a fiber at low **a** and high **b** magnification. Cross sections of hollow (**c**, **d**), folded ribbon **e** and solid **f** fibers. Reproduced with permission from Ref. [19]. Copyright 2005, Wiley–VCH

Unlike the LC spinning method, polymer-free spinning process using dilute, low-viscosity CNT dispersions (0.6 wt%) as spinning dope. By adjusting the pH (pH > 13 or pH < 1), polymer-free, surfactant-stabilized CNTs were flocculated/coagulated rapidly and uniformly to afford continuous CNTs gel fibers (Fig. 3a, b) [19]. In addition, hollow fibers, folded ribbon fibers, and solid CFs can also be easily achieved by this method (Fig. 3c–f) [19]. Due to the absence of polymer, the electrical conductivities (15 S cm^−1^) of these fibers were two orders of magnitude higher than that of fibers made by polymer-based coagulation spinning method (0.2 S cm^−1^).

#### Dip-Coating Method

**Fig. 4:**
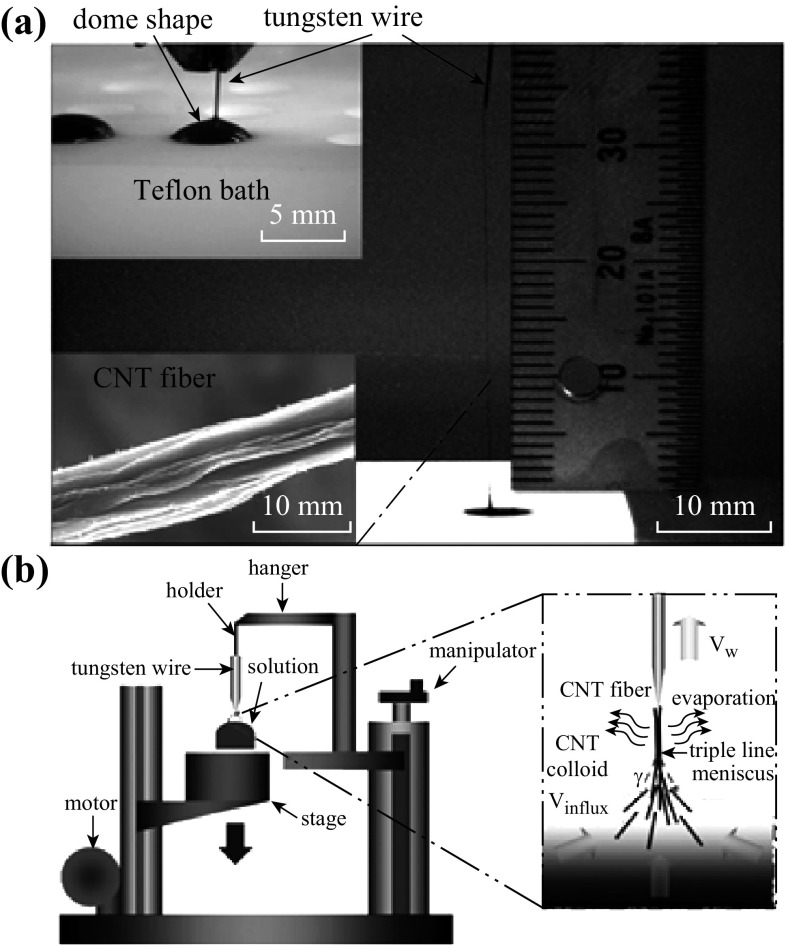
**a** Optical photograph showing the tungsten wire immersed in a SWNT colloidal solution (upper inset; scale bar 5 mm), the SWNT fiber being formed during the withdrawal process (main panel; scale bar 10 mm). SEM image of the lower inset showing the magnified SWNT fiber in the dotted circle (scale bar 10 mm). **b** Schematic of the experimental setup and the formation mechanism of the SWNT fiber. Reproduced with permission from Ref. [20]. Copyright 2009, Wiley–VCH

Besides the above three spinning techniques, a quite simple dip-coating method of fabricating CFs without any additive was developed with no additional electrical equipment or complex apparatus. This method utilizes microfluidics, which includes capillary condensation, capillary flow, and surface tension, and results in the self-assembly and self-alignment of SWNT colloids (Fig. 4) [20]. The resulting SWNT fibers with a diameter of 10–20 μm and a length of several tens of centimeters possess good electrical conductivity of 3000 S cm^−1^.

### Solid-State Spinning Approaches

#### Spinning 1D CFs from CNT Array

**Fig. 5:**
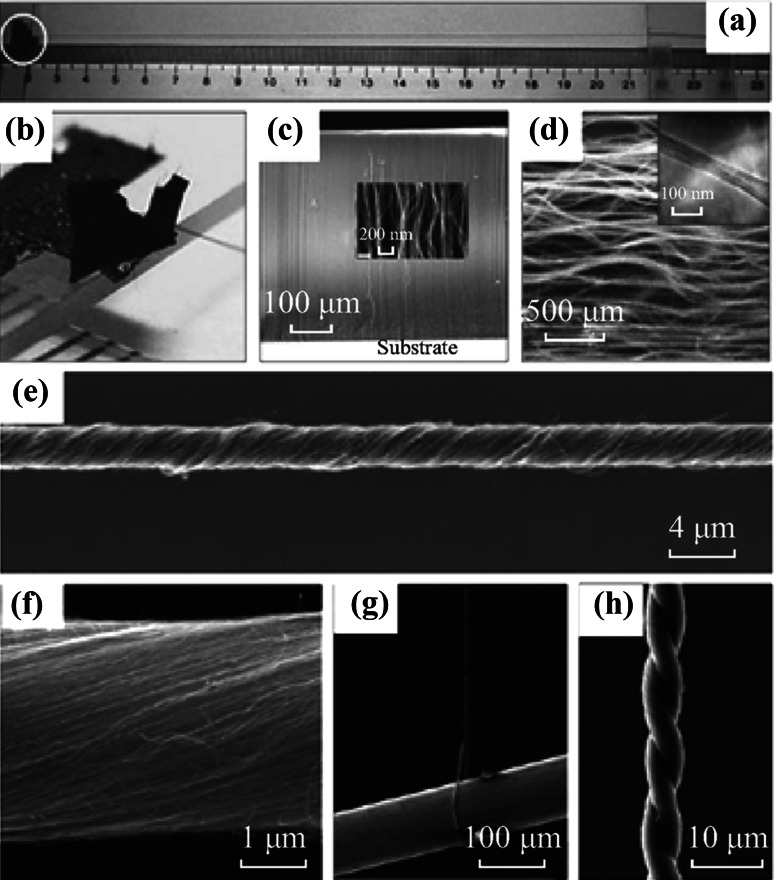
**a** A CF pulled out from a freestanding CNT array, **b** magnified images and SEM images **c** of CNT array, **d** SEM image of the yarn of **a**, transmission electron microscope (TEM) image of a single thread of the CFs shown in inset, SEM images of CF at low **e** and high **f** magnification, **g** CF forming a loop around a Ni wire and then twisted, **h** SEM image of a two-ply twisted fiber. **a**–**d** Reproduced with permission from Ref. [21]. Copyright 2002, Nature Publishing Group. **e**–**h** Reproduced with permission from Ref. [23]. Copyright 2007, Wiley–VCH

Drawing CNTs out from superaligned CNT array is an efficient way to prepare long CFs. The original work was done by Fan and co-workers. They produced a continuous yarn of pure CNTs (Fig. 5a) by pulling out a bundle of CNTs from a CNTs array several hundred micrometers high and grown on a silicon substrate. Figure 5b shows a 100-μm-high, freestanding CNT array with an indentation region that is being turned into a 30-cm-long and 200-μm-wide yarn. It was estimated that an array area of roughly 1 cm^2^ can generate about 10 m of yarn. To achieve continuous yarns, the CNTs in the arrays must be superaligned and held together by van der Waals interactions to form bundles (Fig. 5c). The as-spun yarns were composed of parallel threads that have diameters in the range of several hundreds of nanometers (Fig. 5d) [21].

Inspired by this report, Zhu’s group has done a series of work in the fabrication of CFs with high strength [22–26]. They synthesized a series of CNT arrays with lengths up to 4.7 mm by an ion-beam assisted deposition technique. The CNT arrays were spinnable in a wide range of lengths (0.5–1.5 mm), much longer than those reported previously [22]. Such long arrays rendered CFs with superior strength and electrical conductivity, and the highest *σ* of 3.3 GPa for CFs were spun from the 1-mm array [22]. The properties of CFs can be also improved by decreasing the diameter of the fibers or increasing the packing density of CNTs in the fiber (Fig. 5e, f) [23, 24]. As a result, the CFs can be twisted to hold a 0.1-mm-diameter Ni wire (Fig. 5g, h).

In addition, the effect of chemical post-treatments was also demonstrated. The covalent bonding of gold nanoparticles to the CFs remarkably improves conductivity, whereas annealing CFs in hydrogen-containing atmosphere leads to a dramatic decrease in conductivity [25]. Besides common CNT constitution, well-aligned, pearl-like CNTs were also assembled into macroscopic fibers. Due to the unique morphology, these fibers possess tensile strength of 350 MPa and electrical conductivity of 250 S cm^−1^ [26].

#### Direct Spinning Based on Chemical Vapor Deposition (CVD) Technique

**Fig. 6:**
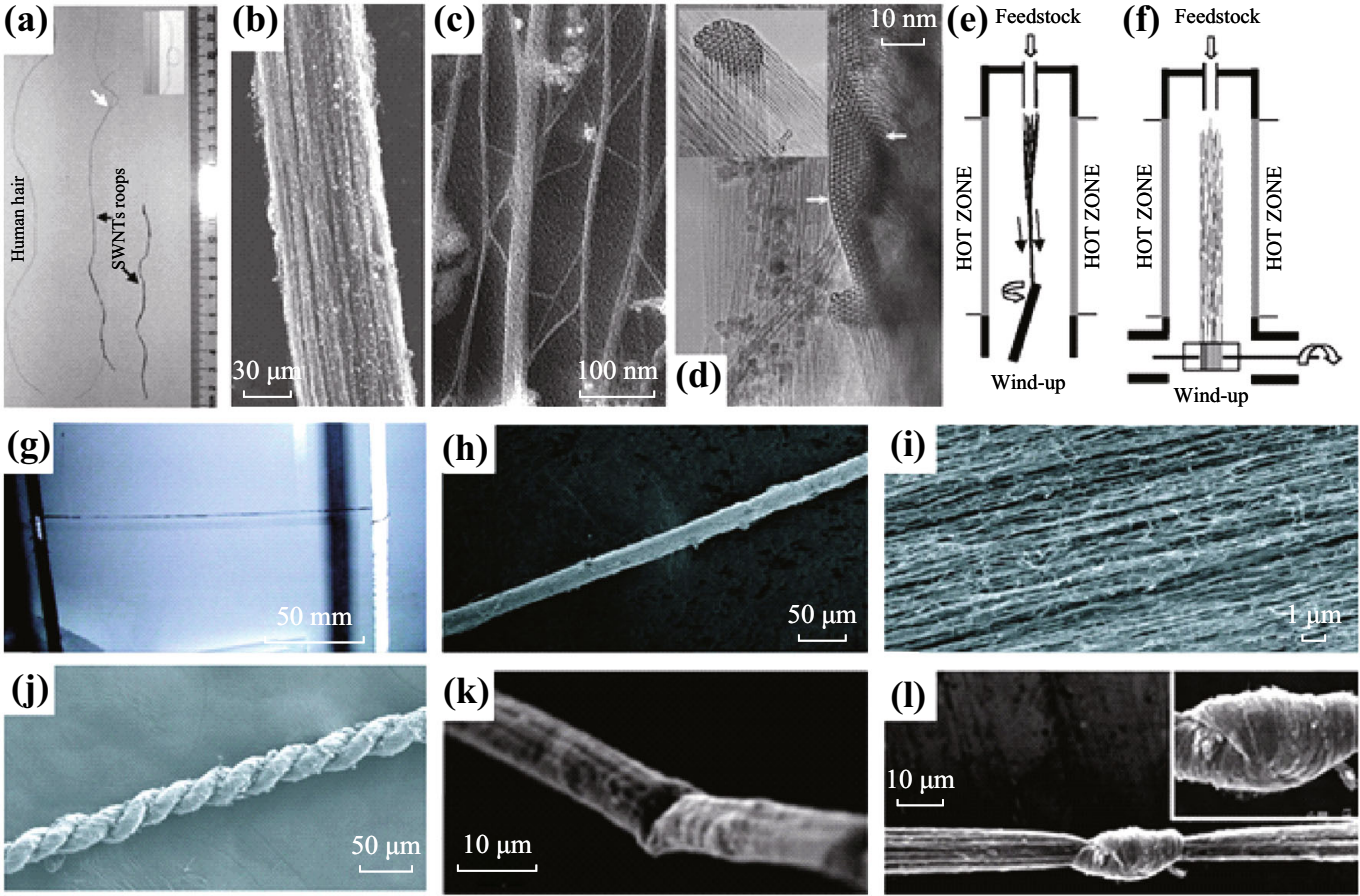
**a** Optical image showing a human hair and two as-grown SWNT fibers. SEM images of CF at low **b** and high **c** magnification. **d** HRTEM image of a top view of a SWNT fiber. Schematic of the direct spinning process with the spindle aligned at 25° (**e**) and normal to **f** the furnace axis. **g** Photograph of CFs wound from the spindle (left) onto a second spindle (right). SEM images of CF at low (**h**) and high **i** magnification. **j** A twisted CF after its removal from the furnace. SEM image of CFs showing a kink induced by bending (**k**) and showing an overhand knot (**l**). **a**–**d** Reproduced with permission from Ref. [27]. Copyright 2002, American Association for the Advancement of Science. **e**–**j** Reproduced with permission from Ref. [28]. Copyright 2004, American Association for the Advancement of Science. **k**, **l** Reproduced with permission from Ref. [29]. Copyright 2010, Wiley–VCH

CVD technique is a common approach to synthesize CNTs. In 2002, the CVD technique was modified to directly synthesize microscopic 1D CFs. Long CNT strands (20 cm in length, a diameter of 0.3 mm, Fig. 6a, b) consisting of aligned SWNTs (Fig. 6c) were achieved through the catalytic pyrolysis of n-hexane with an enhanced vertical floating technique [27]. The edges of the spun SWNT strands were smooth, with a few individual nanotube bundles protruding out of the edge (Fig. 6b). High-resolution transmission electron microscopy (HRTEM) images (Fig. 6d) show that each bundle is composed of aligned SWNTs.

This method was then carried forward by Windle’s group [28–31]. They drew the CVD-grown CNT aerogel onto a rotating rod, produced continuous fibers with a degree of twist (Fig. 6e–i). This endowed the CNT fibers with excellent flexibility. The fibers can be unwound from the spindle and wound up onto another rod and can hold the twisting state after removal from the rod (Fig. 6j) [28]. Because the structure of the fibers itself consists of a network of smaller filament subunits (nanotubes and bundles), they can bend through very tight radii without apparent permanent damage and showed knot efficiencies of 100% (Fig. 6k, l) [29]. By optimizing the process conditions including using three different hydrocarbons, changing the concentration of iron nanocatalyst, the maximum *σ* of CFs was reached to 1.46 GPa [30].

Based on these prior works, Li et al. further advanced this approach and fabricated novel continuous CFs with a multilayered structure. The CFs, featured with a length of over several kilometers and a quality close to conventional textile yarns, can be controlled to be either hollow or monolithic with compacted or detached CNT monolayers by controlling the spinning process [32].

#### Twisting CNT Films

**Fig. 7:**
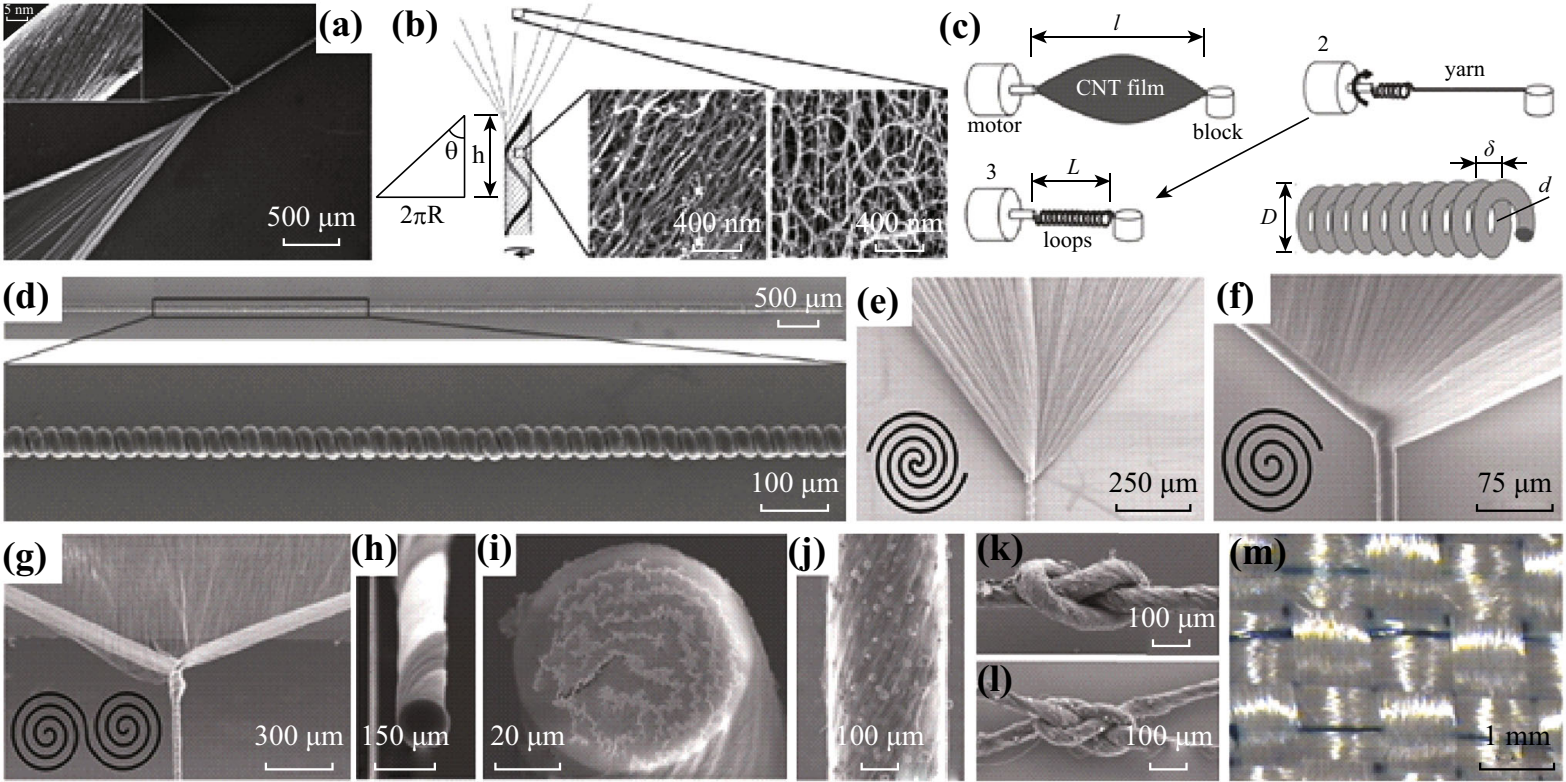
**a** SEM image of a 200-nm-thick CF made from a CNT film with a width of 2 mm. **b** The schematic image left illustrates the twisting process induced elongation for the film and the two SEM images right come from the unstrained and strained part of the film, respectively. **c** Illustration of the spinning process of spring-like CNT ropes. **d** SEM images of a 4.4-mm-long section of rope consisting of highly uniform, perfectly arranged loops. SEM images of Fermat **e**, Archimedean **f** and dual Archimedean **g** type scrolls, which are illustrated in the insets, respectively. **h**, **i** SEM images of Si_3_N_4_NT@MWNT biscrolled yarn. The brighter areas correspond to MWNTs. **j** SEM image of TiO_2_@MWNT yarn. SEM images of overhand knot in 95% LiFePO_4_@MWNT yarn **k** and carrick bend knot between two 88% SiO_2_@MWNT yarns (**l**). **m** Photograph of 85% TiO_2_@MWNT yarn sewn into Kevlar textile. **a**, **b** Reproduced with permission from Ref. [33]. Copyright 2009, Wiley–VCH. **c**, **d** Reproduced with permission from Ref. [36]. Copyright 2012, Wiley–VCH. **e**–**m** Reproduced with permission from Ref. [40]. Copyright 2011, American Association for the Advancement of Science

Twisting CNT thin films with mesh-like structure can afford uniform CF (Fig. 7a). Although this method may not be suitable for continuous production of CFs, the control of the diameters and twisting degrees is more convenient [33–35]. Since the twisting process led to the stretch and alignment of nanotubes (Fig. 7b), the interbundle junctions were reinforced and the fibers have higher strengths than the films [33]. Twisting the fibers to a higher degree, a yarn-derived spring-like CNT rope, that consisted of uniform, neat loops with perfect arrangement over long distance, were formed (Fig. 7c, d) [36]. The elongation at break point of these CNT ropes is up to 285% by loop opening and straightening during elongation. Within a moderate strain (20%), the CNT rope behaved like a spring with a stable spring constant when stretched by 1000 times with energy absorption during contraction [36–38]. This twisting–spinning process was further employed to produce a highly twisted yarn-derived double-helix structure. The double-helix configurations were stable and consisted of two single-helical yarn segments, with controlled pitch and much larger strain up to 150% [39].

Besides the spinning of neat CFs, this method can make the powders and nanofibers spinnable. Combined CNT sheets with other functional materials, more than 95 wt% of powders or nanofibers were incorporated into scrolled CNT yarns, while maintaining the guest functionality in the meantime (Fig. 7h–j). The complex structures of scroll sacks were related to twist-dependent extension of Fermat spirals (Fig. 7e), Archimedean spirals (Fig. 7f), or spiral pairs (Fig. 7g) into scrolls. Although a very small amount of CNT host is employed to mechanically confine the unbonded powder guest, biscrolled yarns can still be knotted and sewn (Fig. 7k–m). This technology was used to make yarns of superconductors, lithium-ion battery materials, graphene ribbons, catalytic nanofibers for fuel cells, and titanium dioxide for photocatalysis [40].

#### Other Methods

**Fig. 8:**
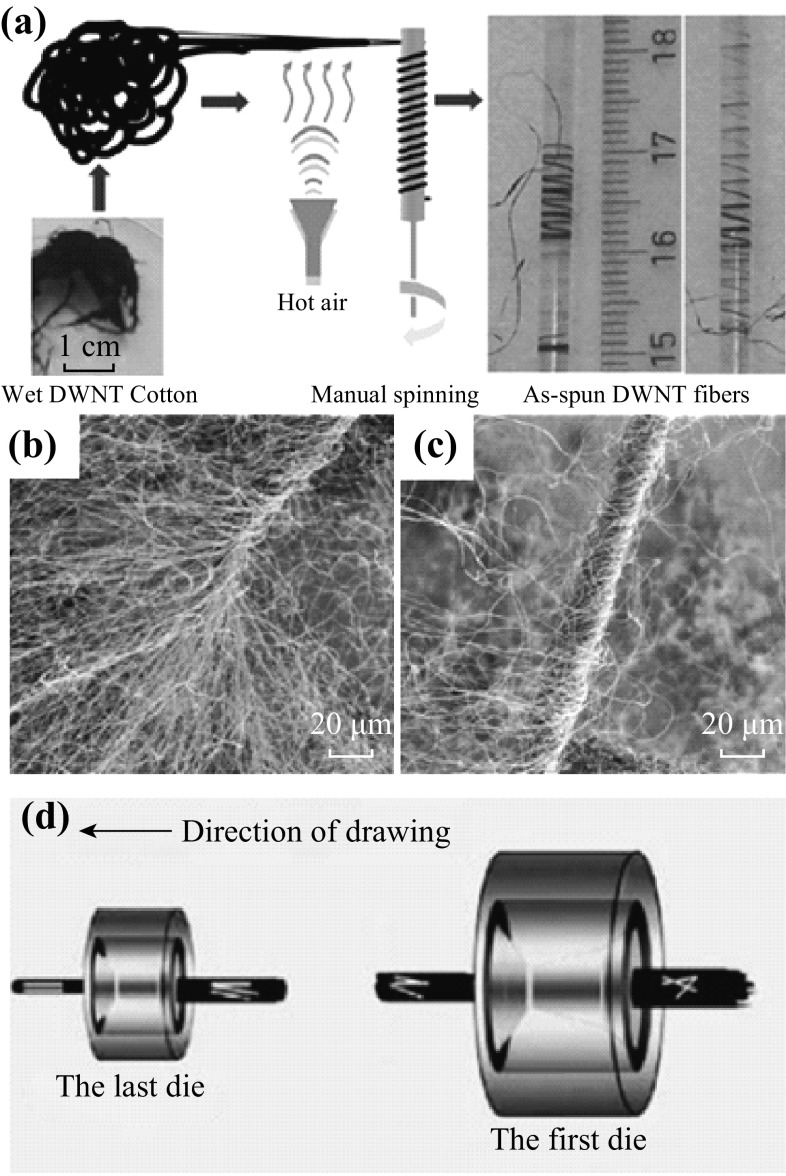
**a** Schematic illustration of the drawing–drying spinning process and optical images of the as-spun DWNT fibers. **b**, **c** SEM images of CNT cotton based fibers. **d** Schematic of fiber shaping by drawing CNT films through a series of diamond wire drawing dies. **a** Reproduced with permission from Ref. [41]. Copyright 2007, Wiley–VCH. **b**, **c** Reproduced with permission from Ref. [42]. Copyright 2007, Wiley–VCH. **d** Reproduced with permission from Ref. [44]. Copyright 2008, American Chemical Society

Different from the method of direct spinning based on CVD technique, which involves the directly drawing and twisting of the fresh generated CNT hot aerogel, Ci et al. fabricated continuous CFs from the cool DWNT cotton based on surface-tension-driven densification. The starting, cotton-like material must be wet in order to draw a continuous fiber because the wet material is denser and has stronger internanotube interactions [41]. Figure 8a shows a schematic illustration of the spinning process, and an optical image of the as-spun DWNT fibers. Similarly, a 35-mm-long quartz-supported CNT cotton was spun into 10-cm-long fibers with good mechanical strength through simply pulling and rotating [42]. Individual CNTs in the cotton were easily collected and aligned along the pulling direction during fiber spinning (Fig. 8b, c).

Electrophoretic approach is generally used in the field of assembly. The early try of assembling CNTs into fibers was finished by Alldredge and co-workers. A carbon fiber tip was withdrawn slowly from a dispersion of SWNT under an electric field. During this process, CNTs were gathered around and assembled one-by-one at the positively charged carbon fiber tip, yet only 5-cm-long fibers could be achieved [43]. Distinct from the former assembly method, Liu et al. invented a step-by-step way to prepare CNT yarns by drawing the CVD-grown films through a series of diamond wire drawing dies (Fig. 8d). Passing through the series dies with decreased pore diameters, the CNT networks were highly aligned and densified into 1D fibers [44, 45].

No matter what methods applied, the nanotubes in CF must be superaligned and densely packed to achieve high performance. Based on the above, we can draw a conclusion that shear rate within the extrusion orifice, flow-induced alignment in the direction of the fluid velocity and the spontaneous ordering in the liquid crystalline phase are all responsible for the alignment. Polarized Raman spectroscopy and XRD can been used to quantitatively probe the degree of alignment. Meanwhile, high-speed drawing, twisting, and surface-tension-driven densification can increase in interbundle contact and packing density.

We will give an example to show how to improve the degree of alignment and packing density by adjusting process parameters. One typical work by Windle’s group systematically explored the effects of density and orientation on the properties of CNT fibers [31]. The degree of alignment and density can be increased simultaneously by increasing the fiber-winding rate, which is a basic precept for the processing of commercial polymer fiber. Additionally, running the fiber through a vapor stream can also significantly densify the fiber due to the surface tension effect. Another important processing technique to improve the mechanical performance is decreasing the diameters of the fiber, which will effectively reduce the flaws along the fiber samples according to Griffith size-scaling law.

## Graphene Fibers: 1D Graphene Macroscopic Architectures

Inspired by the assembly strategies for CFs, 1D GFs were also assembled through analogous approaches such as LC spinning, twisting their 2D films, electrophoretic method. Even so, many novel methods were further developed to achieve GFs with high strength and high electrical conductivity.

### Liquid-State Spinning Approaches

**Fig. 9:**
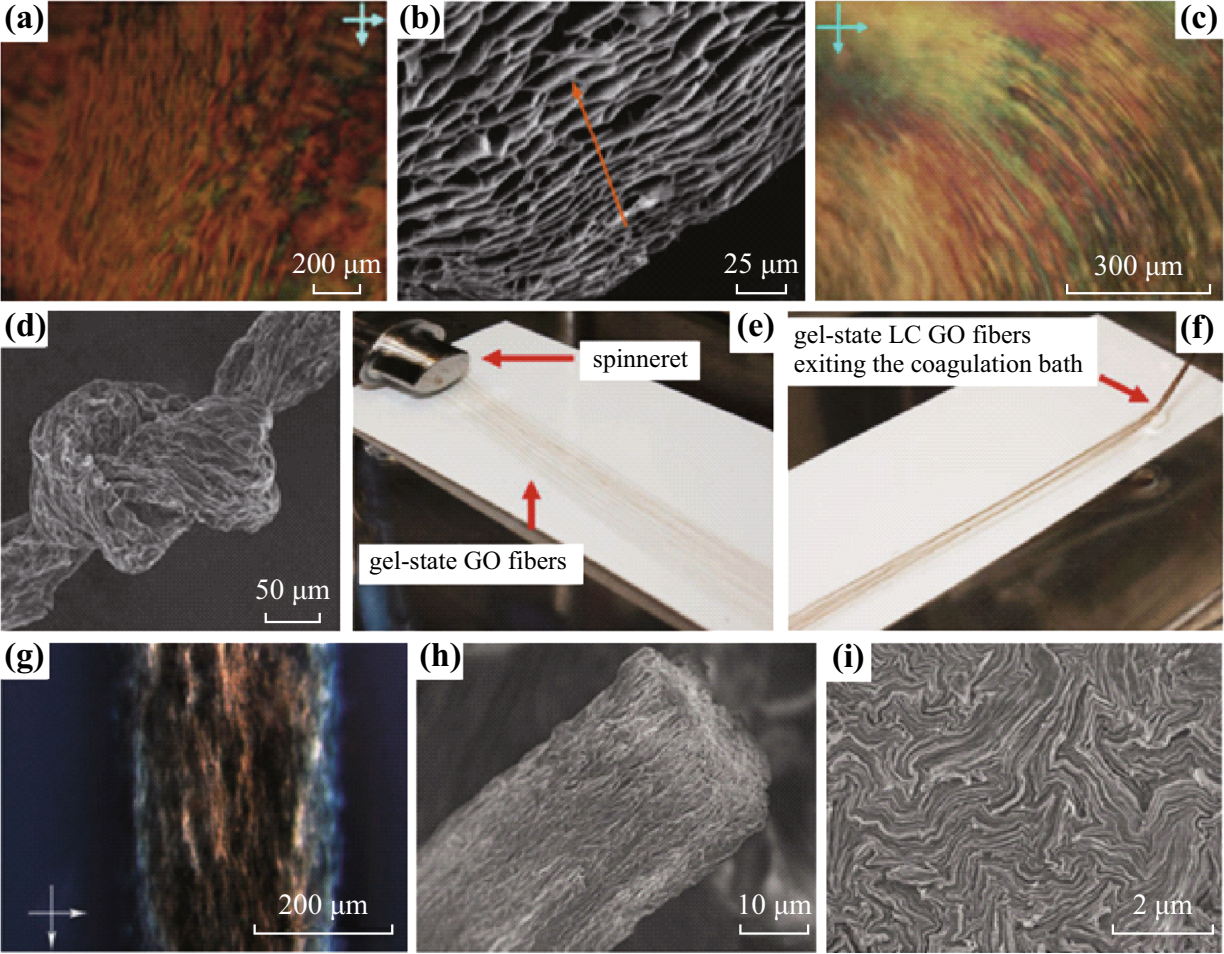
**a** POM observation of GO aqueous dispersions with a fingerprint texture of chiral phase. **b**, **c** Cryo-SEM images and POM textures for GO chiral LCs. **d** a tight knot of GF. **e**, **f** photographs of GO yarns produced using a multi-hole spinneret. **g** POM observation of gel-state GO fiber showing birefringence. SEM images of a GO fiber **h** and its magnified cross section (**i**). **a**–**d** Reproduced with permission from Ref. [46]. Copyright 2011, Nature Publishing Group. **e**–**i** Reproduced with permission from Ref. [49]. Copyright 2013, Wiley–VCH

Graphene or graphene oxide (GO) can yield LC phase due to its high aspect ratio (or width/thickness ratio) and sufficient dispersibility/solubility with the solution concentration up to 30 mg mL^−1^ [46–49]. This LC phase is promising for processing of neat graphitic macroscopic articles such as fibers. In 2011, Gao’s group firstly spun continuous GF utilizing the liquid crystal phase behavior of GO sheets in aqueous solution (Fig. 9a) [46]. The gel-state graphene was extruded from glass syringes and injected into the NaOH/methanol solution under 1.5 MPa held by N_2_ flow. The sheet alignment inherited from the intrinsic lamellar order of LCs provides strong interactions between contacted sheets that were responsible for the strong mechanical strength of GFs. The locally crumbled structures of individual sheets made the GFs flexible (Fig. 9b, c), which can be even woven into a compact knot (Fig. 9d) [46]. The GFs were then functionalized by MnO_2_ and polyaniline and used as electrodes of yarn supercapacitors [50, 51].

**Fig. 10:**
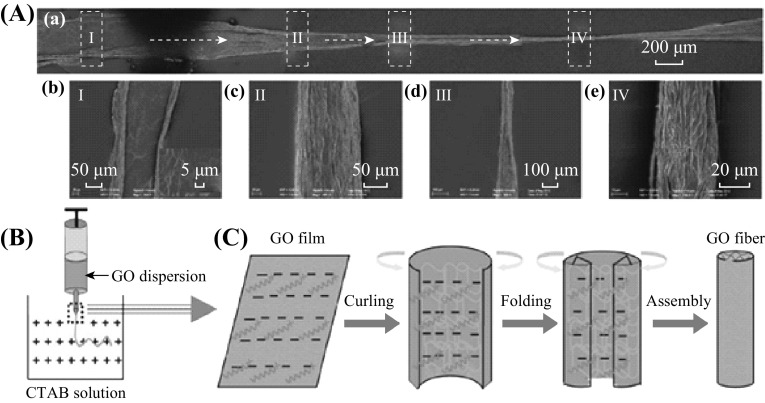
**A** SEM images illustrating the assembly process of the GO fiber. Schematic illustration of the apparatus **B** and assembly mechanism **C** of wet-spinning GO fibers. Reproduced with permission from Ref. [52]. Copyright 2012, Nature Publishing Group

Wallace’s group used a multi-hole spinneret to produced GO yarns (Fig. 9e, f). They revealed the correlation of processability with LC behavior (Fig. 9g), aspect ratio, and the dispersion concentration to provide a viable platform for spinning of LC GO [49]. By adjusting the GO sheet size and polydispersity, the very low concentrations GO dispersions (as low as 0.075 wt%) can be used as spinning dope to prepare the continuous GFs [49]. The morphology of the fiber reveals that GO sheets were stacked in layers with some degree of folding and were orientated along the fiber axis direction (Fig. 9h, i). Neat and macroscopic GFs with mechanical strength of 182 MPa and electrical conductivity of 3500 S m^−1^ were also be spun by Yu’s group. They injected GO dispersion into CATB solution, and the curliness-fold formation mechanism of GO fibers was proved by SEM observations (Fig. 10) [52]. By introducing shear stress during wet-spinning process, flat and uniform ribbons with high flexibility were also produced [53].

**Fig. 11:**
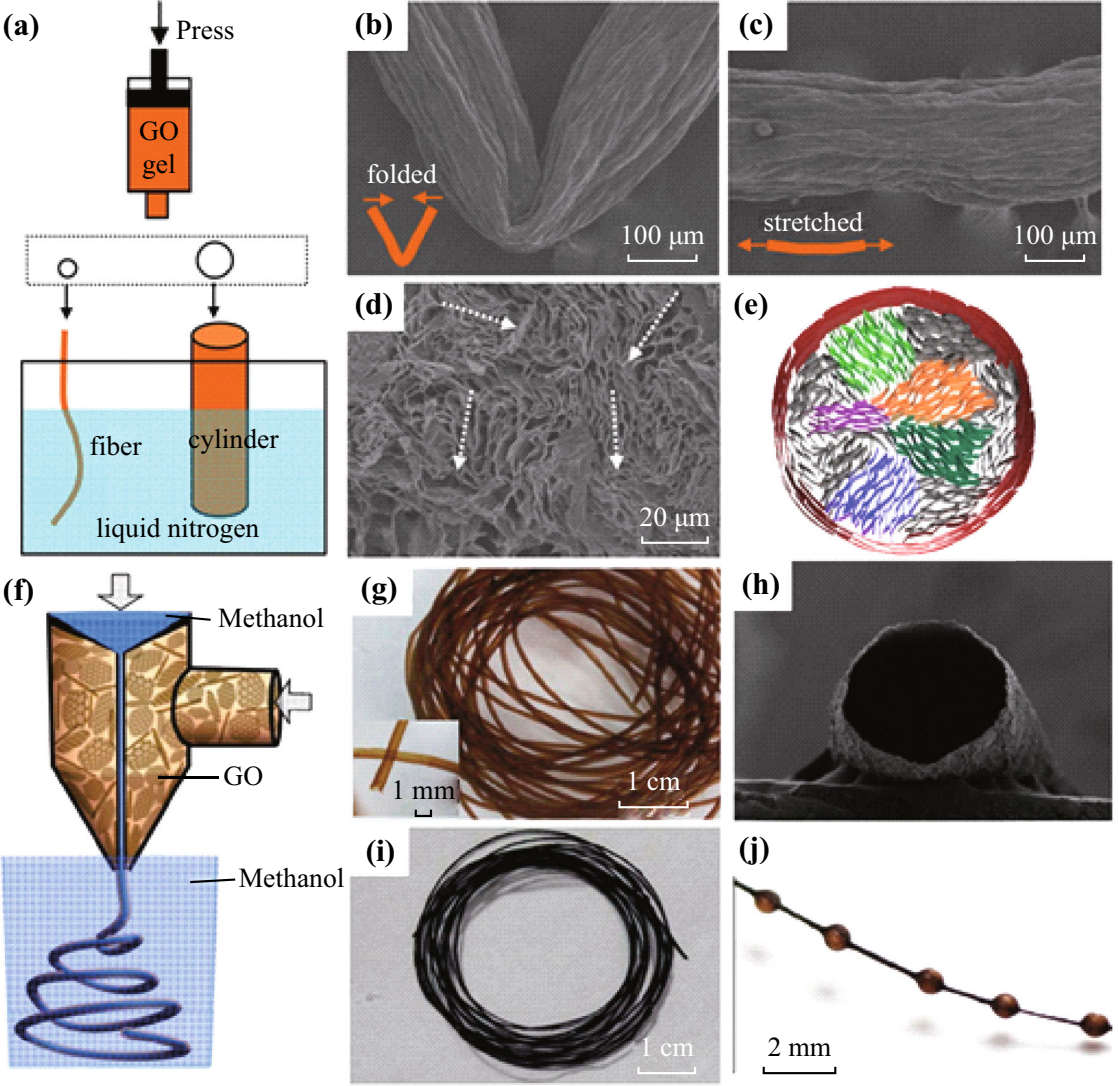
Scheme for preparation of GO porous fibers **a** and hollow fibers (**f)**. SEM images of folded **b**, stretched **c** and fracture morphology **d** of GPFs. **e** Schematic core–shell structure model for GPFs. Photographs of GO-HFs in coagulation bath **g** and the naturally dried GO-HFs (**i**). **h** SEM images of naturally dried GO-HF. **j** A Photograph of necklace-like GO-HF. **a**–**e** Reproduced with permission from Ref. [54]. Copyright 2012, American Chemical Society (**f**–**j**). Reproduced with permission from Ref. [55]. Copyright 2013, American Chemical Society

Neat GO aerogel fibers with unique “porous core-dense shell” structure from flowing GO LCs with lamellar ordering were fabricated by the combination of spinning technology and ice-templating strategy (Fig. 11a) [54]. Graphene porous fibers (GPFs) were achieved after chemical or thermal reduction, which were flexible and can be folded (Fig. 11b). The folded GPFs was stretched to its original straight shape without any fracture (Fig. 11c). The SEM observation revealed that graphene sheets interconnect with each other and form axially aligned empty cells (Fig. 11d, e). The GPFs held high *σ* and high compression strength [54].

Neat, morphology-defined, graphene-based hollow fibers were fabricated via a coaxial two-capillary spinning strategy [55]. The coaxial two-capillary spinneret was fabricated by inserting a stainless steel needle (connected to a syringe-containing coagulation bath) into a branched glass tube (filled with GO suspension) with a capillary tip. Figure 11f shows a schematic illustration of the apparatus for spinning with the coagulation bath of methanol solution containing 3 M KCl. The semitransparent graphene oxide hollow fibers (GO-HFs) (Fig. 11g) were continuously produced, and the hollow structure was reflected by the open tip (Fig. 11g, inset) and SEM observation (Fig. 11h). After being naturally dried, the GO-HFs were converted to black color (Fig. 11i). Replacing the inner fluid of KCl/methanol solution with compressed air, the obtained GO-HFs displayed a necklace-like structure (Fig. 11j).

**Fig. 12:**
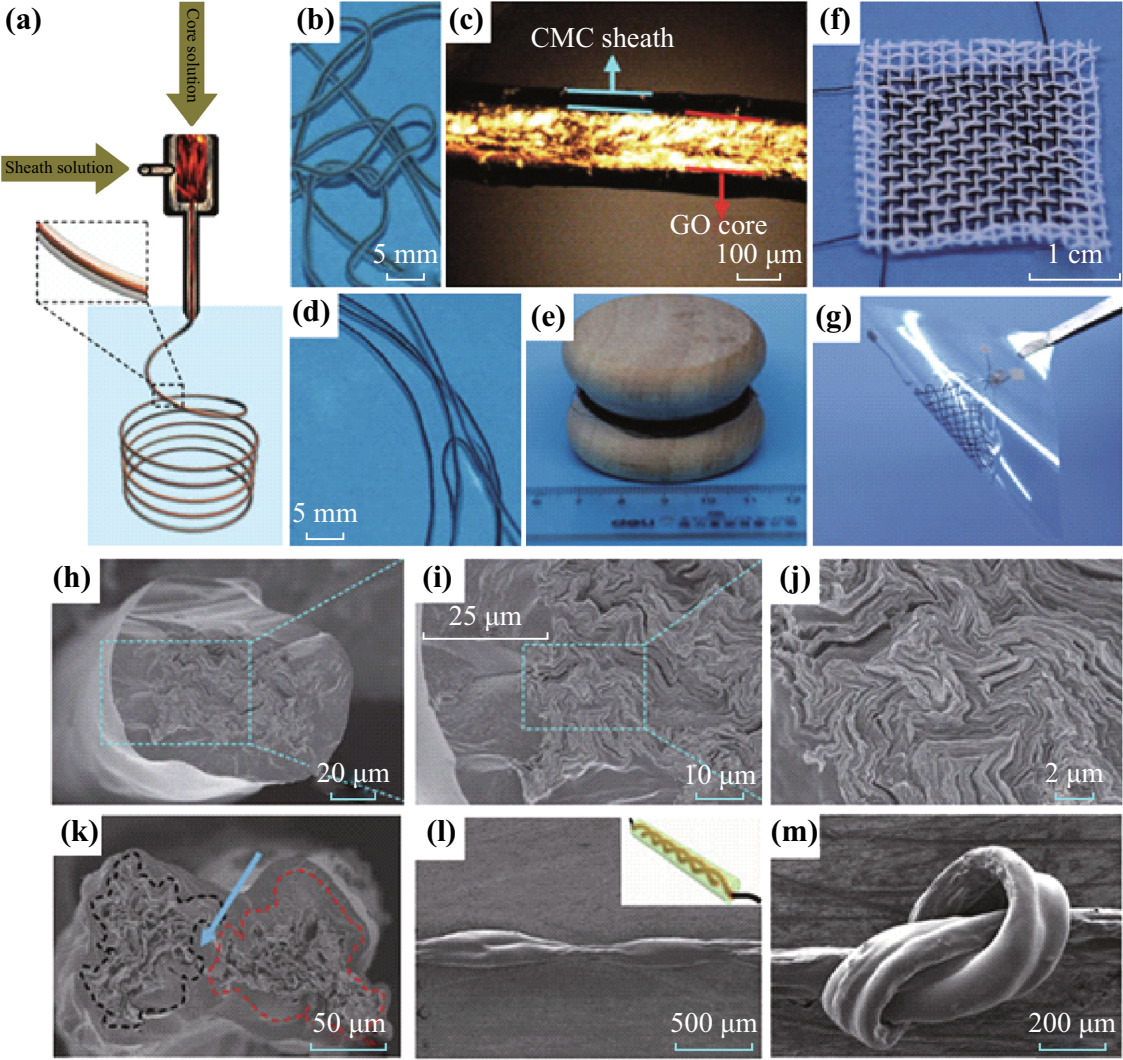
**a** Schematic illustration showing the coaxial spinning process. The magnified image of wet GO@CMC fiber **b** and GO@CMC fiber (**d**). **c** POM image of wet GO@CMC fiber indicating the core-sheath structure and the well-aligned GO sheets in the core part. **e** The macroscopic photograph of the RGO@CMC coaxial fiber. **f** Two intact coaxial fibers woven with cotton fiber. **g** Supercapacitor device based on the cloth fabricated by two coaxial fibers. **h**–**j** SEM images of coaxial fibers. SEM images of cross-sectional **k** and side **l** view of a two-ply YSC. Inset of **i** shows the schematic illustration of YSC. **m** SEM image of a two-ply YSC knot. Reproduced with permission from Ref. [56]. Copyright 2014, Nature Publishing Group

This coaxial wet-spinning technology is further extended to prepare core-sheath GFs [56]. The spinning process is shown in Fig. 12a. GO dispersion and sodium carboxymethyl cellulose (CMC) aqueous solutions were chosen as inner and outer spinning dopes, respectively. The as-prepared coaxial fibers were featured with GO as core and CMC as sheath (Fig. 12b, c), and the CMC sheath wrapped the core tightly without any gaps and void (Fig. 12h–j). Treated by chemically reduced with hydroiodic acid, the color of the core was changed from brown to dark while the polyelectrolyte sheath retained well (Fig. 12d). A 100-m-long coaxial fiber was achieved by this approach (Fig. 12e). The integration of high flexibility and scalable fabrication made these coaxial fibers promising in co-woven with cotton yarns (Fig. 12f). A cloth supercapacitor was then fabricated by using two individual 40-cm-long coaxial fibers as anode and cathode (Fig. 12g). Besides the cloth supercapacitor, the yarn supercapacitors were also prepared by tightly intertwining two coaxial fibers followed by coating a layer of H_3_PO_4_-PVA gel electrolyte as ion source (Fig. 12k–m). The as-prepared yarn supercapacitors were very safe, and short circuit never happened in the assembly and working process of YSCs because of the protection of the CMC sheath.

Chemical functionalization of graphene with polymer can enhance the dispersibility in specific solvents and afford them LC behavior. Polyacrylonitrile (PAN) and poly (glycidyl methacrylate) (PGMA) chains were covalently and uniformly grafted onto GO surfaces via a simple free radical polymerization process [57, 58]. The continuous, conductive, flexible graphene composite fibers were then spun utilizing them as building blocks.

**Fig. 13:**
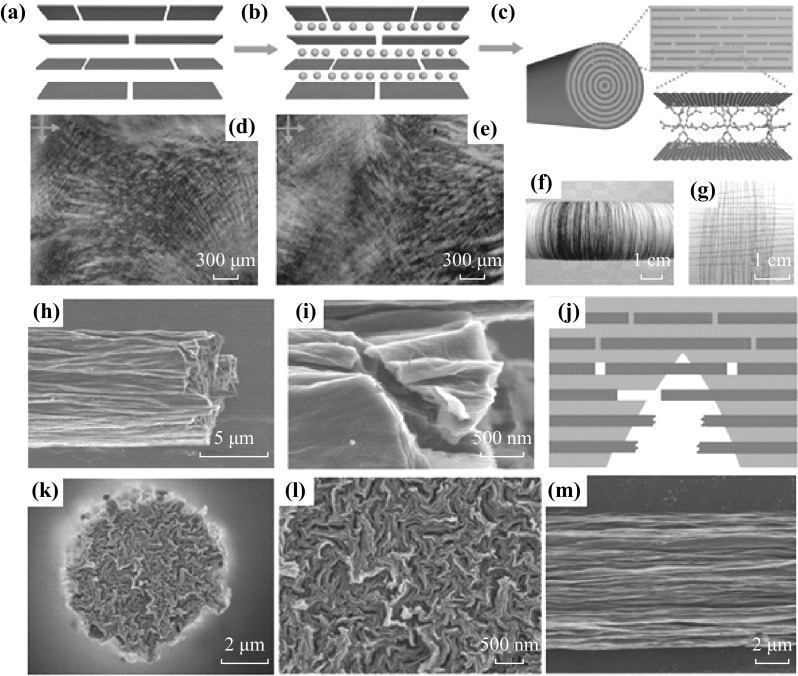
Schematic illustration of LCST strategy **a**–**e** and the resulting composite fibers (**f**, **g**). Morphology of fracture section of GO-HPG fibers **h**, **j** and deformation mechanism model under tensile. SEM images of the cross section **k**, **l** and lateral view **m** of a GO-SA fiber. **a**–**j** Reproduced with permission from Ref. [60]. Copyright 2014, Nature Publishing Group. **k**–**m** Reproduced with permission from Ref. [62]. Copyright 2016, Springer

To make the multifunctionalization of graphene-based fibers fast and easy, Gao’s group combined liquid crystal self-templating (LCST) approach and wet-spinning technology to continuously fabricate biomimetic composites fibers (Fig. 13a–g). They introduced hyperbranched molecules (HPG) [59, 60] into the LC-state GO dispersion to achieve nacre-mimic fibers with excellent mechanical performance (*σ* = 652 MPa, *E* = 20.9 GPa). A deformation mechanism model was also proposed based on the SEM observation (Fig. 13h–j). Besides HPG, PVA [61] and sodium alginate (SA) [62] acted as guests were also mixed with GO host. The assembled macroscopic GO-SA composite fiber inherited the alignment of the GO sheets from the LC phase (Fig. 13k–m), reached 784.9 MPa and 58 GPa for *σ* and *E*, respectively. Apart from the above macromolecules, inorganic components such as Ag nanowires [63], bismuth oxide nanotubes [64] and montmorillonite [65] were also employed as introduced guests to endow graphene-based composite fibers with multiple functions such as high electrical conductivity, improved electrochemical properties and flame retardant.

### One-step Dimensionally Confined Hydrothermal Strategy

Besides the above wet-spinning method, Qu’s group developed a one-step dimensionally confined hydrothermal strategy to prepared GFs [66]. They injected aqueous GO suspension into the glass pipeline with a 0.4-mm-inner diameter by use of a syringe and baked it at 230 °C for 2 h after sealing up the two ends of the pipeline. The wet state GF matching the pipe geometry was produced. It was released from the pipeline by flow of N_2_ and was hold by glass slides for drying in air [66]. To endow the fiber multifunction, they integrated the GFs with Fe_3_O_4_ and TiO_2_ and obtained functional GFs with magnetic and photoelectric (electrical current induced by light) response [66]. The Fe_3_O_4_ functionalized GFs was then acted as both substrate and catalyst for CVD growth of CNTs. The subsequent 1D CNTs and 2D graphene hybrid fibers with a large surface area (ca. 79.5 m^2^ g^−1^) and high electrical conductivity (1200 S m^−1^) were achieved [67]. This strategy is then extended to using PP pipeline instead of glass pipeline and vitamin C, and low temperature chemical reduction replace of hydrothermal reduction [68].

### Electrophoretic Self-Assembly Method

Reduced GO nanoribbon (GONR) fibers were also fabricated by using an electrophoretic self-assembly method without any polymer or surfactant. Specifically, a graphitic tip as a positive electrode was immersed into the chemically reduced GONR colloidal solution in a Teflon vessel containing the counter electrode. The distance between two electrodes is ca. 5 mm, and the fiber is drawn out under a constant voltage ranging from 1 to 2 V. After annealing treatment, the fiber showed superior field emission performance with a low potential for field emission and a giant field emission current density when compared with vertically aligned few-layer graphene and single-layer graphene films prepared by electrophoretic deposition [69].

### Self-Assembly from 2D Graphene Films

Different from the above three methods using GO as precursors, self-assembly method directly utilizes graphene films to fabricate GFs. The 1D fiber-like structures were self-assembled from 2D graphene films in an organic solvent (e.g., ethanol, acetone) and then dried to give the porous and crumpled structure [70, 71]. The morphology and pore structure of the fibers can be adjusted by controlling the evaporation of solvents with suitable surface tension [72–74].

## Mechanical Properties of CNTs/Graphene Fibers

### Mechanical Properties of CFs

Based on the assembly approaches, the mechanical properties of CFs show a broad range from 109 to 8800 MPa for *σ* and 1.25 to 397 GPa for *E* (as shown in Table 1). In general, CFs spun from CNT aerogel/array show higher *σ* and *E* than those produced by wet-spinning approach. To further improve the mechanical performance to compete with conventional high-performance fiber materials (e.g., Kevlar and CFs), it is fundamentally important to probe the critical factors affecting the mechanical properties of CFs. The CNT structures (diameter, aspect ratio, defects, morphology, purity, and orientation) [75] and CF geometries (e.g., diameter, twist angle, and volume fraction) are two major factors dominating CFs’ mechanical behavior. In the case of CNT structures, SWNTs or DWNTs are the best candidates to achieve good fiber performances compared with MWNTs. The well-aligned CNT arrays yield fibers with much higher performance, while wavy and entangled arrays give poor properties [76]. The larger the aspect ratio of graphene units and CNTs, the higher the tensile strength of the CF. In the case of fiber geometries, both the *σ* and *E* would reduce with increasing twisting MWNT fibers to a larger extent [77].

**Table 1:** Summary of the preparation methods, and mechanical properties of the most relevant CFs

Fiber type	Year	Preparation method	Strength (MPa)	Modulus (GPa)	References
CFs	2000	Wet-spinning approach, coagulated in PVA solution	150	15	[6]
	2002	Wet-spinning approach, coagulated in PVA solution, stretched	230	40	[92]
	2003	Wet-spinning approach, SDS surfactant, PVA solution coagulation, post process with acetone-washing bath	1800	80	[9]
	2004	Wet-spinning approach, DNA-stabilized dispersions	109	14.3	[7]
	2004	Wet-spinning approach, fuming sulfuric acid suspensions	116 ± 10	120 ± 10	[18]
	2004	Spinning from a CVD reaction zone	100–1000		[28]
	2005	Wet-spinning approach, polymer-free flocculation-based process, coagulation by adjust the pH of water	770	8.9	[19]
	2005	Wet-spinning approach, hot-drawing treatments	1400–1800	45	[11]
	2005	Spun from an aerogel formed by CVD	1460	10–30	[30]
	2006	Modified coagulation spinning, no surfactant,	500	5–10	[93]
	2006	Spun from an array of 4.7 mm CNT	3300		[22]
	2007	Drawing-drying process from CNT cotton	299	8.3	[41]
	2007	Spun from CNT aerogel	8800	397	[31]
	2007	Spun from an array of longer nanotubes			[25]
	2007	Spun from an CNT array, twisted	1350–3300	100–263	[23]
	2007	Spun from an CNT array, twisted	1910	330	[24]
	2007	Spun from an CNT cotton	180		[24]
	2008	Wet-spinning approach based on lyotropic liquid crystalline phase	150 ± 6	69 ± 41	[16]
	2008	Wet-spinning approach, DNA as binder		1.25	[8]
	2008	Spun from an CNT array	1240		[26]
	2010	Spun from CNT array	1300	95	[76]
	2011	Spun from CNT array	2500 ± 310	120 ± 23	[80]
	2013	Wet-spinning CNT liquid crystals	1000 ± 200	120 ± 50	[94]
	2014	Spun from an aerogel formed by CVD	3760–5530		[95]
	2015	Spun from an aerogel formed by CVD	1000		[96]
	2016	Twist-spun CNT yarns	2000	170	[97]
	2017	Twisting as-grown SWNT film	3300–3700	30–80	[98]
Graphene fibers	2011	Wet-spinning approaches	140	7.7	[46]
	2012	Wet-spinning approaches	182	8.7	[52]
	2012	One-step dimensionally confined hydrothermal strategy, treated at 800 °C	420		[66]
	2012	Wet-spinning approaches, HPG grafted	125	8.2	[59]
	2012	Wet-spinning approaches, CNT, PVA grafted	580	400	[79]
	2013	Wet-spinning approaches, GO LC template, HPG crosslink	652	20.9	[60]
	2013	Wet-spinning approaches, large-sized GO	360	12.8	[55]
	2013	Wet-spinning approaches, large-sized GO	442	22.6	[49]
	2013	Wet-spinning approaches, PVA grafted	199	17.1	[61]
	2013	Wet-spinning approaches, PAN grafted	452	8.31	[57]
	2013	Wet-spinning approaches, large-sized GO	214	47	[86]
	2013	Wet-spinning approaches, large-sized GO, Ca^2+^ crosslink	501	11.2	[87]
	2013	Wet-spinning approaches, PGMA grafted	500	18.8	[58]
	2014	Coaxial wet-spinning approaches	116		[56]
	2014	One-step dimensionally confined hydrothermal strategy, vitamin C reduced	150	1.9	[68]
	2015	Wet-spinning approaches, montmorillonite	270	44	[65]
	2015	Wet-spinning approaches, 30% small-sized GO, high-temperature graphitization	1150	135	[88]
	2016	Wet-spinning approaches, SA	784.9	58	[62]
	2016	Wet-spinning approaches, high-temperature graphitization	1450	282	[89]

Owing to the weak van der Waals interaction between the neighboring CNT, the CNT can easily slide on each other, resulting in a low *E* of CFs. To solve this problem, strong interaction between the tubes must be introduced. Electron beam irradiation is an effective approach to cause stable links between neighboring CNT within bundles. The mechanical properties showed a 30-fold increase in the bending modulus due to the formation of stable crosslinks that effectively eliminate sliding between the nanotubes [78].

Besides the electron-beam-induced crosslinking approach, a method based on filtration-twisting was proposed to fabricate CNT-based composite fibers [35]. The strength values for epoxy- and PVA-infiltrated fibers were increased to 0.9–1.6 and 0.7–1.3 GPa, while modules were improved to 30–50 and 20–35 GPa, respectively. The toughness of PVA-infiltrated fibers reaches 50 J g^−1^, far superior to commercially used high-strength fibers [35]. Further increased toughness results from combining CNTs and reduced GO flakes in solution-spun polymer fibers. The gravimetric toughness approaches 1000 J g^−1^, far exceeding spider dragline silk (165 J g^−1^) and Kevlar (78 J g^−1^) [79].

**Fig. 14:**
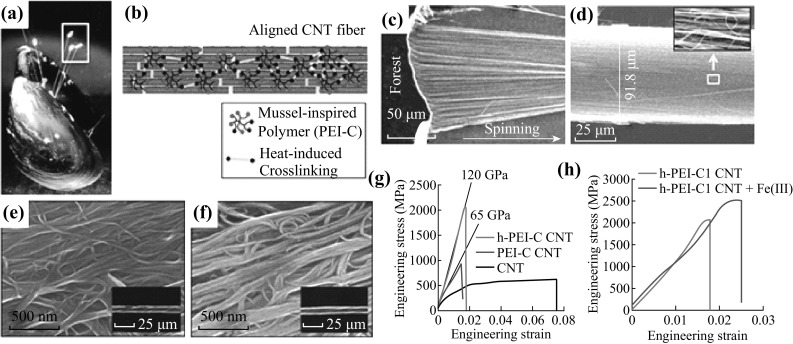
**a** Photograph of a mussel attached to Si substrate. **b** A schematic illustration of one-dimensionally aligned CFs reinforced by mussel-mimetic adhesives. SEM observations of fiber spinning process **c** and the spun CFs (**d**). High-magnification SEM images of the densified CF **e** and h-PEI-C treated CF **f**. Mechanical curves of the CFs. (**g**, **h**) Stress–strain curves of h-PEI-C-CNT (*red*) and h-PEI-C-CNT + Fe(III) (*blue*). Reproduced with permission from Ref. [80]. Copyright 2011, Wiley–VCH. (Color figure online)

Apart from PVA or epoxy, super-strong CFs with tensile strength up to GPa scale were also fabricated inspired by the molecular mechanics of mussel adhesive formation (Fig. 14a, b). The CFs were spun from the CNT forest (Fig. 14c, d). Treated with twisting, infiltrating the adhesive polymer poly(ethylenimine) catechol (PEI-C), and solvent evaporation, the strong and densified fibers (Fig. 14e, f) were prepared. The PEI-C-infiltrated CFs (PEI-C-CNT) exhibited 65% increase in *σ* from 0.55 to 0.91 GPa compared with the pure fibers. After crosslinking at 120 °C for 2 h, *σ* and *E* of the fibers increased to 2.2 GP and 120 GPa, respectively (Fig. 14g). Besides the heat-induced crosslinking, the Fe-catechol coordination and Fe-induced oxidative crosslinking chemistry further enhanced *σ* to 2.5 GPa and toughness of 32.5 MJ m^−3^ (Fig. 14h) [80].

### Mechanical Properties of Graphene Fibers

The first GFs show a *σ* of ca. 140 MPa with breakage elongation of 5.8%, but has good flexibility to make tight knots [46]. Compared to the intrinsic nature of graphene (*σ*, 130 GPa, *E*, 1.1 TPa) [81–83], a plenty of work should be done to improve the mechanical performance of GFs. Based on the subsequent study of GFs, the mechanical performance can be enhanced from three aspects: (1) improving the alignment of graphene sheets along the fiber axis; (2) decreasing the structural defects that include sheets boundaries, voids and impurities; and (3) enhancing the interlayer interaction of the constituent graphene sheets by either covalent or noncovalent bonds [84, 85]. The stretching operation leads to a high alignment of GO sheets and benefit for the formation of compact structures [86]. When draw ratios were increased from 1.09 to 1.45, the *σ* is raised from 124 to 214 MPa and *E* reaches 47 from 14 GPa. To decreasing the structural defects, large-sized GO were used as building block [49, 55, 86, 87]. As a result, the improved *σ* ranged from 214 to 501 MPa. To enhance the interlayer interaction, PVA [61], HPG [59], PAN [57], PGMA [58], and SA [62] were introduced into interlayer galleries. The resultant fibers exhibited *σ* of 199, 652, 452, and 500 MPa, *E* of 17.1, 20.9, 8.31, and 18.8 GPa, respectively (as shown in Table 1).

High-temperature graphitization process is a more effective method to achieve high-performance GFs since the structural defects were removed or restored. The *σ* break through 1 GPa for the first time. In 2015, Lian’s group improved the mechanical performance of GFs to 1.15 GPa by combining the large-sized graphene with small-sized graphene, aided by flowing-stretching and high-temperature graphitization treatment [88]. Recently, Xu et al. raised this value to 2.2 GPa through a full-scale synergetic defect engineering method to minimize the possible defects at all levels ranging from atomic to macroscale, the best mechanical performance until now [89–91].

## Concluding Remarks and Prospects

Many methods are developed to assemble CNTs and graphene into macroscopic fibers. For CNT fibers, on account of the entanglement nature between individual nanotubes, the approach of drawing CNT out from superaligned CNT arrays or aerogels is promising in achieved continuous, high-strength and high-electrical-conductive fibers. For GFs, considering the excellent dispersity of GO in water and organic solvent, liquid-state spinning approaches may be more competitive in the future.

Although great progress has been made to improve the mechanical performance of macroscopic CFs and GFs, a wide gap still exists considering the intrinsic properties of CNT and graphene building blocks. Until now, the *σ* of CFs and GFs both exceed 1 GPa, comparable to that of the universal carbon fibers. In order to improve the performance of macroscopic CFs and GFs, defects such as voids and disordered orientation should be minimized.

To further upgrade the combined performance of CFs and GFs, especially the mechanical strength and electrical conductivity, much work is waiting to be explored and more sophisticated understandings about the relationship between the structure and performance is of significant important from the viewpoints of both fundamental research and real application. On the one hand, we should establish a real-time system to monitor the structural evolution of the basic CNTs/graphene units during the fiber-forming process, which will contribute to the structure controlling of the fibers. On the other hand, the processing techniques (e.g., stretching, fining, densifying, annealing) used to enhance the performance of polymeric fibers and carbon fibers should be borrowed to optimize the fabrication process throughout.
